# Bayesian AEWMA control chart under ranked set sampling with application to reliability engineering

**DOI:** 10.1038/s41598-023-47324-0

**Published:** 2023-11-16

**Authors:** Imad Khan, Muhammad Noor-ul-Amin, Dost Muhammad Khan, Umair Khalil, Emad A. A. Ismail, Uzma Yasmeen, Bakhtiyar Ahmad

**Affiliations:** 1 Department of Statistics, Abdul Wali Khan University Mardan, Mardan, Pakistan; 2 Department of Statistics, COMSATS University Islamabad, Lahore Campus, Lahore, Pakistan; 3 Department of Quantitative Analysis, College of Business Administration, King Saud University, P.O. Box 71115, Riyadh, 11587 Saudi Arabia; 4 Department of Mathematics & Statistics, BROCK University, St. Catharines, Canada; 5 Higher Education Department Afghanistan, Kabul, Afghanistan

**Keywords:** Engineering, Materials science, Mathematics and computing

## Abstract

The article introduces a novel Bayesian AEWMA Control Chart that integrates different loss functions (LFs) like the square error loss function and Linex loss function under an informative prior for posterior and posterior predictive distributions, implemented across diverse ranked set sampling (RSS) designs. The main objective is to detect small to moderate shifts in the process mean, with the average run length and standard deviation of run length serving as performance measures. The study employs a hard bake process in semiconductor production to demonstrate the effectiveness of the proposed chart, comparing it with existing control charts through Monte Carlo simulations. The results underscore the superiority of the proposed approach, particularly under RSS designs compared to simple random sampling (SRS), in identifying out-of-control signals. Overall, this study contributes a comprehensive method integrating various LFs and RSS schemes, offering a more precise and efficient approach for detecting shifts in the process mean. Real-world applications highlight the heightened sensitivity of the suggested chart in identifying out-of-control signals compared to existing Bayesian charts using SRS.

## Introduction

Variation plays a vital role in the operation of the manufacturing industry and control charts are extremely valuable and effective tools in Statistical Process Control (SPC) practice. These charts are widely utilized in different service subdivisions to monitor production process. The key aim of applying control charts is to ensure process constancy and to identify rare deviations in production so that the control system can implement necessary corrections before non-conforming items are produced. Walter A. Shewhart suggested the concept of memoryless charts in the 1920s which, with later modifications, served as the basis for modern Statistical Process Control (SPC). Page^1^ suggested the cumulative sum (CUSUM) chart, whereas the exponentially weighted moving average (EWMA) control chart was presented by Roberts^2^. These CUSUM and EWMA charts are predominantly effective at detecting small to medium shifts in the manufacturing process. On the other hand, the Shewhart chart excels at being able to identify significant fluctuations in the process parameters. Alduais et al.^3^ introduces a Rayleigh EWMA scheme, enhancing the detection capabilities of the traditional V_SQR_ chart for monitoring process variability in Rayleigh distributed processes, evaluated through design limits, parameters, and Monte Carlo simulations. To address this issue, Capizzi and Masarotto^4^ proposes an AEWMA chart to address the limitations of traditional EWMA control charts, aiming for improved performance in detecting both small and large shifts in the mean of a sequence of independent observations. Comparisons with different control charts demonstrate that AEWMA schemes provide more balanced protection against shifts of varying sizes. Other authors, including^5–9^ have also made significant contributions to this research area. Haq et al.^10^ introduced an AEWMA chart to monitor the production process. This control chart utilizes estimated shift size, ARL and SDRL as performance measurement tools. Zaman et al.^11^ introduced an innovative adaptive version of the EWMA chart that incorporates Tukey's bi-square functions. This approach has shown particular effectiveness in detecting shifts during the monitoring of the process mean. Atif et al.^12^ proposed a new configuration for the AEWMA chart intended for process mean surveillance. The chart's effectiveness is evaluated based on the ARL and SDRL metrics. Abbas et al.^13^ focus on the application of RSS in designing dispersion charts for manufacturing processes, emphasizing their effectiveness over traditional methods in detecting process variations. The study highlights the improved performance of the proposed control chart structures, illustrating their timely identification of special causes in a bottle-filling process. Abbas et al.^14^ introduce a novel scheme for dispersion charts using neoteric RSS, enhancing accuracy in quality control and environmental monitoring. The comparison analysis confirms the enhanced effectiveness of dispersion charts based on NRSS in the context of a non-isothermal continuous stirred tank chemical reactor model. Mohammadkhani et al.^15^ studied extensively reviewed the application of RSS for control chart design, seeking to bridge research gaps and offer suggestions for future research in statistical process monitoring (SPM) and control chart design.

The previously mentioned research on statistical quality control charts has mostly concentrated on the traditional approach, which relies exclusively on available sample data and does not use prior knowledge. Bayesian estimation offers an alternative approach to estimate a population parameter, incorporating both sample and prior information to revise the posterior (P) distribution for an unknown population parameter. Girshick and Rubin^16^ proposed the notion of a quality control chart within the Bayesian framework. Saghir et al.^17^ suggested a Bayesian chart for the P distribution that takes into account both informative and non-informative priors. They also used LFs to track the production process. Bourazasa et al.^18^ studied a Bayesian method for online monitoring, prioritizing outlier detection and leveraging predictive distribution across diverse data sets. Simulation results emphasized its advantages over frequentist methods, showcasing robustness to prior sensitivity and model misspecification, with practical examples highlighting its suitability for short production runs and online phase I monitoring. Asalam and colleagues^19^ proposed an improved modified EWMA chart using Bayesian analysis. The evaluation of the suggested chart considers the ARL and SDRL, demonstrating its superior capability in promptly identifying out-of-control signals compared to existing charts. Noor and co-authors^20^ explored a Bayesian hybrid EWMA control chart suitable for P and PP distributions, examining informative and non-informative priors, along with various LFs. The evaluation of the chart's performance was conducted based on the ARL and SDRL. Noor et al.^21^ conducted a study on the AEWMA chart using Bayesian theory for identifying the process mean, utilizing various LFs. The assessment of the control chart’s performance commonly entails the examination of the run length profiles. Lin et al.^22^ proposed an EWMA chart using Bayesian methodology for identifying changes in the process variance within a distribution-free process. They identified the favorable sampling properties of the statistic proposed in their study, designed for monitoring the time-varying process distribution. Moreover, they demonstrated the efficacy of the control chart through an extensive simulation study. Hybrid EWMA chart utilizing Bayesian concept that employs various RSS designs along with an informative prior for monitoring the location parameter was suggested by Khan et al.^23^. The performance of the recommended chart was appraised using run length results, and a comparison was made with hybrid EWMA (HEWMA) and AEWMA charts applying Bayesian concepts applying SRS. Liu et al.^24^ presented a Bayesian AEWMA control chart employing diverse LFs and PRSS for accurate process mean shift detection, outperforming other control charts, especially under PRSS schemes. Its efficacy was verified in semiconductor manufacturing, confirming its dominance in identifying out-of-control signals over existing methods. Wang et al.^25^ examined the impact of ME on the Bayesian EWMA control chart, evaluating various RSS designs and loss functions. By utilizing Monte Carlo simulations and actual data, the research illustrates the notable impact of ME on the control chart, particularly endorsing the median RSS approach in such scenarios.

Ranked set sampling is a sampling technique that involves ordering the units in a sample according to their responses. It has advantages over SRS, such as reducing the sample size and increasing efficiency. By combining Bayesian inference with the control chart and RSS strategies, the chart becomes more robust and sensitive to changes in the process. This combination allows for better decision-making regarding process control and facilitates timely corrective actions. Overall, the role of Bayesian chart using RSS designs is to enhance the accuracy and efficiency of process monitoring, improve quality control, and support effective decision-making in statistical quality management. Hence, the primary objective of our research is to propose an AEWMA control chart using a Bayesian methodology that integrates various RSS designs, such as median RSS (MRSS) and extreme RSS (ERSS). Furthermore, we integrate informative prior distributions into symmetric and asymmetric LFs, such as SELF and LLF, applicable to both the P and PP distributions. To assess the performance of the offered chart. We analysis various metrics, such as ARL and SDRL.

The remaining part of the article is structured as follows: The second section introduces the Bayesian method and LFs. Section “Ranked set sampling” discusses the various RSS systems. Section “Proposed Bayesian AEWMA control chart” presents a Bayesian AEWMA control chart with multiple RSS systems. Section “Simulation study” includes a comparison between the suggested and existing charts, and Section “Results discussion and main findings” presents the key results. Section “Real data applications” contains the numerical analysis, while Section “Conclusion” concludes the article. Section “Limitations of the study” and “Future recommendation” include the study limitations and recommendations, respectively.

## Bayesian approach

The Bayesian approach is a statistical methodology that utilizes probability theory to update our beliefs about the likelihood of a hypothesis when new evidence is provided. It involves starting with a prior probability distribution that represents our initial beliefs regarding the hypothesis and updating it using Bayes’ theorem to obtain a posterior distribution that reflects our revised beliefs. The foundation of the Bayesian approach lies in the recognition that probability serves as a powerful tool for capturing and expressing uncertainty. By leveraging probability, we can seamlessly integrate prior knowledge and uncertainty into our analytical framework. There are two primary categories of prior information: informative prior and non-informative prior. An informative prior is a prior distribution of a parameter that includes pertinent and established knowledge regarding an unidentified population parameter. By integrating existing information, the prior distribution is informed and influenced accordingly. A conjugate prior arises when the prior distribution and the sampling distribution are part of the same family of distributions, enabling simpler analytical calculations and yielding posterior distributions that are of the same form as the prior. The Bayesian approach finds widespread application in diverse fields such as medical research and finance. This approach proves valuable in handling situations characterized by incomplete information and uncertainty. This methodology offers valuable tools for handling and incorporating uncertainty, making it applicable to a broad range of fields. In present study, the variable under study *X* is defined by its mean $\theta$ and variance $\delta^{2}$ with under control process with considering conjugate prior, with parameters $\theta_{0}$ and $\delta_{0}^{2}$, is defined as follows

1 $$p\left( \theta \right) = \frac{1}{{\sqrt {2\pi \delta_{0}^{2} } }}\exp \left\{ { - \frac{1}{{2\delta_{0}^{2} }}\left( {\theta - \theta_{0} } \right)^{2} } \right\}$$

When there is no prior information available about the population parameter, Bayesian statisticians often use a non-informative prior. A non-informative prior is characterized by a prior distribution that has slight influence on the P distribution, aligning with the fundamental principles of the Bayesian approach for integrating preceding information into the analysis. In numerous instances, researchers commonly adopt a non-informative prior that aligns with a uniform distribution. This implies allocating equal probability to all plausible parameter values within a predefined range. The probability function representing the uniform prior distribution is as follows:

2 $$p\left( \theta \right) \propto \sqrt {\frac{n}{{\delta^{2} }}} = c\sqrt {\frac{n}{{\delta^{2} }}}$$

Here *c* represents the constant of proportionality.

The prior function that is proportional to Fisher information matrix was introduced by Jeffrey^26^ because the uniform prior does not satisfy the invariance criterion. The following is Jeffrey's recommended prior probability function:

3 $$p\left( \theta \right) \propto \sqrt {I\left( \theta \right)}$$

Here $I\left( \theta \right)$ represents Fisher information matrix.

For a population parameter theta, the P distribution, which combines a sample distribution and a prior distribution, is mathematizied as:

4 $$p\left( {\theta |x} \right) = \frac{{p\left( {x|\theta } \right)p\left( \theta \right)}}{{\int {p\left( {x|\theta } \right)p\left( \theta \right)d\theta } }}.$$

For a fresh dataset *Y*, the PP distribution, based on the P distribution, is mathematically described as

5 $$p\left( {y|x} \right) = \int {p\left( {y|\theta } \right)p\left( {\theta /x} \right)d\theta } .$$

Bayesian inference heavily relies on the utilization of LFs to address potential risks related with the Bayesian estimator. In the present study, we have incorporated two types of LFs: symmetric (SELF) and asymmetric (LLF). By employing these LFs, we aim to effectively manage and mitigate uncertainties inherent in the Bayesian framework.

### Squared error loss function

The SELF, based on Bayesian theory in the context of estimation, is a mathematical measure used to quantify the discrepancy between the true parameter value and its estimated value. The calculation entails squaring the discrepancy between the estimated parameter value and the actual value. The Bayesian methodology incorporates prior information about the parameter by employing the prior distribution. The SELF is then utilized to assess the quality of the estimate obtained within this framework. By reducing the expected value of the square error loss, Bayesian estimation aims to find the optimal estimate that strikes a balance between the data-driven information and prior beliefs. In the current study, we employed the LF suggested by Gauss^27^. The SELF, which takes into account the variable under consideration *X* and the estimator $\hat{\theta }$ used to calculate the unknown population parameter $\theta$, is mathematized as:

6 $$L\left( {\theta ,\hat{\theta }} \right) = \left( {\theta - \hat{\theta }} \right)^{2}$$

and based on SELF the Bayes estimator mathematically described as:

7 $$\hat{\theta }_{{\left( {SELF} \right)}} = E_{\theta /x} \left( \theta \right).$$

### Linex loss function

Varian^28^ introduced an asymmetric LF called LLF, which is specifically designed to mitigate the risks related with the Bayesian estimator. The LLF can be mathematically expressed as follows:

8 $$L\left( {\theta ,\hat{\theta }} \right) = \left( {e^{{c\left( {\theta - \hat{\theta }} \right)}} - c\left( {\theta - \hat{\theta }} \right) - 1} \right)$$

Utilizing LLF, the Bayesian estimator $\hat{\theta }$ is mathematizied as

9 $$\hat{\theta }_{{\left( {LLF} \right)}} = - \frac{1}{c}InE_{\theta /x} \left( {e^{ - c\theta } } \right).$$

## Ranked set sampling

McIntyre^29^ introduced an innovative sampling scheme known as RSS. This sampling method provides a novel approach for picking a sample from a given population. The complete procedure for implementing RSS can be summarized as follows:

i. Randomly select *m*^2^ independent samples from the target population, and distribute them into *m* sets with an similar size of *m* elements. The ordering of the *m* units within each set can be determined by the researcher's personal judgment, auxiliary variables, or any method that does not require direct measurement.
ii. After arranging all *m* sets, the selection process begins with picking the initial element from the first set, followed by selecting the second element from the second set, and so on. This sequence of actions completes a single cycle of RSS. If required, these two steps can be repeated *r* times to get a sample of size *n* = *rm*. The RSS procedure can be described as follows: $Z_{i(j),r}$, *i*,*j* = 1,2,3,…,*m*; *r* = 1,2,3,…,*c*, where $Z_{i(j),r}$ represents the *j*th order statistic in the *i*th sample set with cycle *r*. The mean and variance of the ranked set sample estimator are presented specifically for the case when c = 1.

Using RSS design, the unbiased estimator for the population mean is mathematically defined as

10 $$\overline{Z}_{{\left( {RSS} \right)}} = \frac{1}{m}\sum\limits_{i = 1}^{m} {Z_{i\left( i \right)} }$$

and with variance

11 $${\text{var}} \left( {\overline{Z}_{{\left( {RSS} \right)}} } \right) = \frac{{\delta^{2} }}{m} - \frac{1}{{m^{2} }}\sum\limits_{i = 1}^{m} {\left( {\mu_{\left( i \right)} - \mu } \right)} .$$

Where μ is overall mean.

### Median ranked set sampling

The median ranked set sampling (MRSS) technique, a modification of the RSS design, was first described by Muttlak^30^. The MRSS estimator was created primarily to increase the accuracy of population mean estimation. The whole process for choosing a sample when utilizing the MRSS design is described below:

i. In the MRSS design, a sample of n units is chosen from the population of interest applying a method akin to RSS. These selected units are subsequently divided into m sets of equal size. Within each set, the units are ordered in ascending fashion utilizing specific variable under consideration.
ii. When the set size is even, the selection process involves picking the smallest units from the two middle elements of the $$\left( {{\raise0.7ex\hbox{$m$} \!\mathord{\left/ {\vphantom {m 2}}\right.\kern-0pt} \!\lower0.7ex\hbox{$2$}}} \right)th$$ set. Likewise, it requires selecting the largest units from the two middle elements of the $$\left( {{\raise0.7ex\hbox{$m$} \!\mathord{\left/ {\vphantom {m 2}}\right.\kern-0pt} \!\lower0.7ex\hbox{$2$}}} \right)th$$ set. In the case where *m* is odd, the selection focuses on the middle elements from the $$\left( {{\raise0.7ex\hbox{${\left( {m + 1} \right)}$} \!\mathord{\left/ {\vphantom {{\left( {m + 1} \right)} 2}}\right.\kern-0pt} \!\lower0.7ex\hbox{$2$}}} \right)th$$ order sets. This sequence of steps completes one cycle of the MRSS scheme. Certainly, this process allows for the repetition of the steps a total of r times, leading to the accumulation of a sample size denoted as *n* = *rm.*

The estimate for the population mean of one cycle in MRSS is defined as follows in a situation of an odd sample size:

12 $$\overline{Z}_{{\left( {MRSS} \right)O}} = \frac{1}{m}\left( {\sum\limits_{i = 1}^{m} {Z_{{i\left( {\frac{m + 1}{2}} \right)}} } } \right)$$

And with variance

13 $${\text{var}} \left( {\overline{Z}_{{\left( {MRSS} \right)O}} } \right) = \frac{1}{m}\left( {\delta_{{\left( {\frac{m + 1}{2}} \right)}}^{2} } \right).$$

The estimator for the population mean using the MRSS design with a single cycle is defined as follows for a sample size that is odd:

14 $$\overline{Z}_{{\left( {MRSS} \right)O}} = \frac{1}{m}\left( {\sum\limits_{i = 1}^{{{\raise0.7ex\hbox{$m$} \!\mathord{\left/ {\vphantom {m 2}}\right.\kern-0pt} \!\lower0.7ex\hbox{$2$}}}} {Z_{{i\left( \frac{m}{2} \right)}} } + \sum\limits_{i = 1}^{{{\raise0.7ex\hbox{$m$} \!\mathord{\left/ {\vphantom {m 2}}\right.\kern-0pt} \!\lower0.7ex\hbox{$2$}}}} {Z_{{\frac{m}{2} + i\left( {\frac{m + 1}{2}} \right)}} } } \right)$$

And with variance

15 $${\text{var}} \left( {\overline{Z}_{{\left( {MRSS} \right)O}} } \right) = \frac{1}{m}\left( {\delta_{{\left( \frac{m}{2} \right)}}^{2} + \delta_{{\left( {\frac{m + 2}{2}} \right)}}^{2} } \right).$$

### Extreme ranked set sampling

Samawi and Muttlak^31^ introduced the concept of the ERSS design. The ERSS design is particularly beneficial in situations where collecting the central unit is more challenging compared to the extreme units. The following outlines are the complete methodology for choosing a sample using the ERSS design:

i. Picked $m^{2}$ units from the underlying population and distribute them into *m* sets of equal size. Rank all the units within each set based on a variable of interest.
ii. If the set size is even, select the smallest units from the $\left( \frac{m}{2} \right)th$ ranked sets and choose the largest elements from the remaining $\left( \frac{m}{2} \right)th$ orderd sets. When m is odd, select the smallest elements from the $\left( {\frac{m - 1}{2}} \right)th$ ranked sets and largest elements from the last $\left( {\frac{m - 1}{2}} \right)th$ ranked sets. The middle unit is selected from the last ranked set.

When the sample size is odd and there is only one cycle, the mean estimator of the ERSS is defined as

16 $$\overline{Z}_{{\left( {ERSS} \right)O}} = \frac{1}{m}\left( {\sum\limits_{i = 1}^{{\left( {\frac{m - 1}{2}} \right)}} {Z_{i\left( 1 \right)} } + \sum\limits_{i = 1}^{{\left( {\frac{m - 1}{2}} \right)}} {Z_{{\left( {\frac{m - 1}{2}} \right) + i\left( l \right)}} } + Z_{{m\left( {\frac{m + 1}{2}} \right)}} } \right)$$

and variance

17 $${\text{var}} \left( {\overline{Z}_{{\left( {ERSS} \right)O}} } \right) = \frac{1}{{2m^{2} }}\left( {\delta_{\left( 1 \right)}^{2} + \delta_{\left( m \right)}^{2} } \right) + \frac{1}{{l^{2} }}\left( {\delta_{{\left( {\frac{m + 1}{2}} \right)}}^{2} } \right).$$

In the situation, when the sample size is odd and single cycle is performed than the mean estimator of ERSS is mathematized as

18 $$\overline{Z}_{{\left( {ERSS} \right)e}} = \frac{1}{m}\left( {\sum\limits_{i = 1}^{{\left( \frac{m}{2} \right)}} {Z_{i\left( 1 \right)} } + \sum\limits_{i = 1}^{{\left( \frac{m}{2} \right)}} {Z_{{\frac{m}{2} + i\left( l \right)}} } } \right)$$

And.

19 $${\text{var}} \left( {\overline{Z}_{{\left( {ERSS} \right)e}} } \right) = \frac{1}{2m}\left( {\delta_{\left( 1 \right)}^{2} + \delta_{\left( m \right)}^{2} } \right).$$

## Proposed Bayesian AEWMA control chart

In this section, we focus on the AEWMA chart constructed using Bayesian theory, investigating the application of diverse RSS strategies for efficient monitoring of irregular variations in the location parameter of a process conforming to a normal distribution. Consider X_1_, X_2_,…, X_n_ as independent and identically normally distributed random variables with a $\theta$ and $\sigma^{2}$. The mathematical description of the probability function is as follows:

20 $$f\left( {x_{t} :\theta ,\sigma^{2} } \right) = \frac{1}{{\sqrt {2\pi \sigma^{2} } }}\exp \left( { - \tfrac{1}{{2\sigma^{2} }}\left( {x_{t} - \theta } \right)^{2} } \right).$$

Let the estimated mean shift ${\widehat{\delta }}_{t}^{*}$ be represented as an AEWMA sequence utilizing $\left\{ {X_{t} } \right\}$. The expression for the estimated mean shift is as follows:

21 $$\hat{\delta }_{t}^{*} = \psi X_{t} + \left( {1 - \psi } \right)\hat{\delta }_{t - 1}^{*}$$

where $\hat{\delta }_{0}^{*} = 0$ and $\psi$ is smoothing constant, for the out-of-control process the estimator $\hat{\delta }_{t}^{*}$ is biased and for the under-control process, the estimator $\hat{\delta }_{t}^{*}$ is unbiased. Haq et al.^10^ introduced the concept of an unbiased estimator for δ in both under control and out-of-control process situations. This unbiased estimator is mathematically described as follows:

22 $$\hat{\delta }_{t}^{**} = \frac{{\hat{\delta }_{t}^{*} }}{{1 - \left( {1 - \psi } \right)^{t} }}.$$

It is offered to use $$\hat{\delta }_{t} = \left| {\hat{\delta }_{t}^{**} } \right|$$ for obtaining estimate of $$\delta$$.

The proposed statistic, denoted as $E_{t}$, is based on the Bayesian theory and incorporates various RSS strategies. It utilizes the sequence $\left\{ {X_{t} } \right\}$ to estimate the process mean.

23 $$E_{t} = f\left( {\hat{\delta }_{t} } \right)\hat{\theta }_{{\left( {RSS_{i} } \right)LF}} + \left( {1 - f\left( {\hat{\delta }_{t} } \right)} \right)E_{t - 1} ,$$

where $i = 1,2,3,$ $$\begin{gathered} RSS_{1} = RSS \hfill \\ RSS_{2} = MRSS \hfill \\ RSS_{3} = ERSS \hfill \\ \end{gathered}$$, $f\left( {\hat{\delta }_{t} } \right) \in \left( {0,\left. 1 \right]} \right.$ and $E_{0} = 0$ such that

24 $$f\left( {\hat{\delta }_{t} } \right) = \left\{ {\begin{array}{*{20}l} {\frac{1}{{a\left[ {1 + \left( {\hat{\delta }_{t} } \right)^{ - c} } \right]}} } &\quad {if \; 0 < \hat{\delta }_{t} \le 2.7} \\ 1 &\quad {if \; \hat{\delta }_{t} > 2.7} \\ \end{array} } \right..$$

Sarwar and Noor-ul-Amin^12^ proposed a function, as illustrated in Eq. (24), that adapts the smoothing constant in response to the estimated shift. The recommended values for the constants used in Eq. (24) are *a* = 7 and *c* = 1 when $1 < \hat{\delta }_{t} \le 2.7$. For $\hat{\delta }_{t}$ values less than or equal to 1, it is suggested to use a value of *c* equal to 2. In this scenario, if the plot statistics exceed the designated threshold ℎ, the process is categorized as out of control. Conversely, if the plot statistics remain below the specified threshold ℎ, the process is considered to be in the state of control.

In instances where both the likelihood function and prior distribution are normally distributed, the resulting posterior distribution also follows a normal distribution, characterized by the mean, θ, and the variance, σ^2^. The probability density function (pdf) is given by:

25 $$P\left( {\theta /x} \right) = \frac{1}{{\sqrt {2\pi } \sqrt {\frac{{\delta^{2} \delta_{0}^{2} }}{{\delta^{2} + n\delta_{0}^{2} }}} }}\exp \left[ { - \frac{1}{2}\left( {\frac{{\theta - \sum\limits_{i = 1}^{n} {\frac{{x_{i} \delta_{0}^{2} + \theta_{0} \delta_{0}^{2} }}{{\delta^{2} + n\delta_{0}^{2} }}} }}{{\sqrt {\frac{{\delta^{2} \delta_{0}^{2} }}{{\delta^{2} + n\delta_{0}^{2} }}} }}} \right)^{2} } \right]$$

where $\theta_{n} = \frac{{n\overline{x} \delta_{0}^{2} + \delta^{2} \theta_{0} }}{{\delta^{2} + n\delta_{0}^{2} }}$ and $\delta_{n}^{2} = \frac{{\delta^{2} \delta_{0}^{2} }}{{\delta^{2} + n\delta_{0}^{2} }}$ respectively.

The $\hat{\theta }_{{\left( {SELF} \right)}}$ for the suggested chart utilizing Bayesian analysis, while accounting for different RSS strategies applying symmetric LF, is depicted as:

26 $$\hat{\theta }_{{\left( {SELF} \right)}} = \frac{{n\overline{x}_{{(RSS_{i} )}} \delta_{0}^{2} + \delta^{2} \theta_{0} }}{{\delta^{2} + n\delta_{0}^{2} }}.$$

The properties of the offered Bayes estimator with SELF *i.e.* $\hat{\theta }_{{\left( {SELF} \right)}}$ is given as $E\left( {\hat{\theta }_{{\left( {SELF} \right)}} } \right) = \frac{{n\theta_{1} \delta_{0}^{2} + \delta^{2} \theta_{0} }}{{\delta^{2} + n\delta_{0}^{2} }}$ and $sd\left( {\hat{\theta }_{{\left( {SELF} \right)}} } \right) = \sqrt {\frac{{n\delta_{{(RSS_{i} )}}^{2} \delta_{0}^{4} }}{{\delta^{2} + n\delta_{0}^{2} }}}$. The Bayes estimator for the recommended chart employing Bayesian theory and applying RSS strategies with the LLF is mathematized as:

27 $$\hat{\theta }_{{\left( {_{LLF} } \right)}} = \frac{{n\overline{x}_{{(RSS_{i} )}} \delta_{0}^{2} + \delta^{2} \theta_{0} }}{{\delta^{2} + n\delta_{0}^{2} }} - \frac{{C^{\prime } }}{2}\delta_{n}^{2} .$$

The properties of $\hat{\theta }_{{\left( {_{LLF} } \right)}}$ is given by $E\left( {\hat{\theta }_{LLF} } \right) = \frac{{n\theta_{1} \delta_{0}^{2} + \delta^{2} \theta_{0} }}{{\delta^{2} + n\delta_{0}^{2} }} - \frac{{C^{\prime } }}{2}$ and $sd\left( {\hat{\theta }_{{\left( {LLF} \right)}} } \right) = \sqrt {\frac{{n\delta_{{(RSS_{i} )}}^{2} \delta_{0}^{4} }}{{\delta^{2} + n\delta_{0}^{2} }}}$ respectively.

Consider a set of feature observations of size *h*, indicated as y_1_, y_2_,…,y_n_. In the suggested AEWMA chart using Bayesian theory and implementing RSS strategies for the posterior predictive distribution, the probability density function of y∣x is presented as:

28 $$p\left( {{\raise0.7ex\hbox{$y$} \!\mathord{\left/ {\vphantom {y x}}\right.\kern-0pt} \!\lower0.7ex\hbox{$x$}}} \right) = \frac{1}{{\sqrt {2\pi \delta_{1}^{2} } }}\exp \left\{ { - \frac{1}{{2\delta_{1}^{2} }}\left( {Y - \theta_{n} } \right)^{2} } \right\}$$

where y∣x normally distributed with $\theta_{n}$ and standard deviation $\delta_{1}$, derived as $\delta_{1} = \sqrt {\delta^{2} + \frac{{\delta^{2} \delta_{0}^{2} }}{{\delta^{2} + n\delta_{0}^{2} }}}$. Using various RSS designs, the estimator of $\theta$ for PP distribution based on LLF defined as

29 $$\hat{\theta }_{LLF} = \frac{{n\overline{x}_{{(RSS_{i} )}} \delta_{0}^{2} + \delta^{2} \theta_{0} }}{{\delta^{2} + n\delta_{0}^{2} }} - \frac{{C^{\prime } }}{2}\tilde{\delta }_{1}^{2}$$

where $\tilde{\delta }_{1}^{2} = \frac{{\delta^{2} }}{k} + \frac{{\delta^{2} \delta_{0}^{2} }}{{\delta^{2} + n\delta_{0}^{2} }}$. The properties of $\hat{\theta }_{LLF}$ is given as $E\left( {\hat{\theta }_{LLF} } \right) = \frac{{n\theta_{1} \delta_{0}^{2} + \delta^{2} \theta_{0} }}{{\delta^{2} + n\delta_{0}^{2} }} - \frac{{C{\prime} }}{2}\tilde{\delta }_{1}^{2}$ and $sd\left( {\hat{\theta }_{LLF} } \right) = \sqrt {\frac{{n\delta_{{(RSS_{i} )}}^{2} \delta_{0}^{4} }}{{\left( {\delta^{2} + n\delta_{0}^{2} } \right)^{2} }}}$ respectively.

## Simulation study

The performance assessment of the offered chart is conducted various different RSS strategies through the utilization of Monte-Carlo simulation method. The run length results are computed for various mean shifts. The in-control process is specified at 370. By adjusting the smoothing constant sci = 0.10, we analyze the impact of the recommended chart with RSS strategies. The following steps outline the simulation process for the proposed method.

### Step 1: setting in-control ARL

i. The prior and sampling distribution are chosen to be the standard normal distribution, and the properties are computed for various LFs. *i.e.,* $E\left( {\hat{\theta }_{{\left( {_{LLF} } \right)}} } \right)$ and $\delta_{LLF}$.
ii. Begin by specifying the hyperparameters for the Bayesian chart. These hyperparameters are vital for defining the prior distribution. For instance, determine the parameters of the prior distribution, which is chosen to be the standard normal distribution, and the associated hyperparameters such as the mean and variance.
iii. Use the specific smoothing constant (e.g., sci = 0.10) as a hyperparameter for determining the threshold value (h). The choice of this hyperparameter influences the sensitivity of the control chart.
iv. Random samples of size n are generated from a normal distribution. Ensure that the hyperparameters of this normal distribution, such as mean and variance, are well-defined as they significantly affect the data generation process.
v. Utilizing Bayesian theory, calculate the AEWMA statistic $E_{t}$, and assess the performance of the process rendering to the proposed design.
vi. In the event that the process is confirmed to be in a state of control, it is advisable to repeatedly move through steps one to three until the moment the process is recognized as being out of control. It is important to document the run-length for the process that remains in control.

### Step 2: for out-of-control ARL

i. The random samples are generated from the normal distribution for the shifted process *i.e.,* $X \sim N\left( {E\left( {\hat{\theta }} \right) + \partial \frac{\delta }{\sqrt n },\delta } \right)$, where $\partial$ represents the shift in the process mean.
ii. Calculate the $E_{t}$ and appraise the process according to the suggested strategy.
iii. If the process is identified as being under control, it is recommended to continue the iterative process of the initial two stages until signals of being out of control are observed. It is essential to keep a record of the run length during the in-control process.
iv. Calculate the ARL and SDRL after 100,000 iterations of steps (i—iii).

## Results discussion and main findings

The comparison of existing chart utilizing Bayesian analysis based on SRS with the offered AWEMA control under different RSS strategies by using two different LFs with the same smoothing constant values shown in Tables 1, 2,3, 4, 5 and 6. Tables 1 and 2 indicates the results for suggested CC utilizing RSS designs (RSS, MRSS, ERSS) and using informative prior based on SELF, and the existing CC using Bayesian theory with SRS for P and PP distribution. The efficiency of the recommended chart is evaluated through $ARL_{1}$, the smaller values of $ARL_{1}$ indicates the fast detection of the out of control motions. According to findings, the proposed AEWMA control chart implemented within RSS strategies exhibits a higher level of sensitivity in identifying out of control signs compared with existing Bayesian chart implemented within SRS. For example, the ARL values of EWMA chart applying Bayesian concept based on SRS with SELF at $\psi = 0.10$ and various shifts *i.e.,* $\sigma$ = 0.0, 0.30, 0.50, 0.80, 1.50, 4 are 371.16, 66.57, 28.35, 13.41, 5.79 and 2.12 and under the similar condition, ARL values of AEWMA chart using Bayesian analysis are 370.16, 43.59, 18.90, 7.90, 2.56 and 1.01. According to the same situation, ARL outcomes for the suggested CC using RSS, MRSS, and ERSS are 370.25, 11.56, 7.41, 3.10, 1.28, 1, and the values under MRSS are 371.55, 15.58, 5.94, 2.57, 1.16, 1 and 370.13, 21.96, 8.30, 3.47, 1.37 and 1are ARL results using ERSS. The results show that the proposed CC, when utilized with RSS designs, exhibits better performance compared with both existing Bayesian EWMA and AEWMA charts implemented with SRS. In a similar vein, when employing the LLF, Table 6 presents a comprehensive comparison among the existing EWMA chart and AEWMA chart implemented with SRS utilizing Bayesian concept, and the proposed chart implemented using RSS strategies with an informative prior. The table provides a detailed analysis of their respective performances and highlights any notable differences or advantages among them. Under SRS, ARL values for EWMA CC at $\psi = 0.25$ and different shifts $\sigma$ = 0.0, 0.30, 0.50, 0.80, 1.50, 4 are 370.23, 103.68, 41.26, 15.79, 5.18 and 1.66. Further, 369.25, 55.67, 27.50, 12.91, 4.08, and 1.08 are the ARL values of AEMWA CC applying SRS. ARL values of suggested CC applying RSS are 371.18, 29.24, 12.42, 5.13, 1.71, 1, under MRSS ARL results are 370.56, 23.96, 9.87, 3.99, 1.43, 1 and ARL results of ERSS are 369.23, 31.48, 13.82, 5.80, 1.88, and 1. The ARL results of the recommended chart, implemented using RSS strategies, demonstrate a significant decrease when larger shifts occur. This suggests that the offered chart is more subtle and effective in identifying out-of-control signs compared to both the existing Bayesian control charts implemented with SRS. The detailed findings of the proposed chart, utilizing RSS strategies, are presented below:

- Based on the observations provided in Tables 1 and 2, it is evident that the run length outcomes for the proposed Bayesian chart, incorporating SELF via RSS stratigies, display a sharp decrease with increasing mean shift. This trend indicates the unbiased nature of the suggested chart. For instance, referring to Table 1 with an ARL of 370 and $\psi$ = 0.10, the ARL results at various shifts such as δ = 0.20 and 0.70 are as follows: 38.40 and 3.93 for RSS, 32.19 and 3.14 for MRSS, and 41.80 and 4.43 for ERSS.
- Similarly, the effectiveness of suggested chart, implemented applying RSS strategies, is appraised applying LLF by altering the values of $\psi$ = 0.10 and 0.25. Tables 3 and 4 display the ARL outcomes of the chart with LLF. The results indicate that with an increase in the value of the weighting constant, the efficiency of the suggested chart diminishes. For example, at $ARL_{0} = 370$, $\psi = 0.10$ and shift δ = 0.20, The corresponding ARL outcomes of the offered chart using RSS, MRSS, and ERSS are 36.50, 31.73, and 41.91. For the same shift δ = 0.20 and $\psi = 0.25$, ARL value for RSS is 50.89, under MRSS is 43.63, and under ERSS is 52.45.
- The findings suggest that with an increase in the value of the weighting constant, the efficiency of the proposed chart diminishes. For instance, with a certain value of the weighting constant, α, and a shift value of δ = 0.20, the corresponding ARL results for the offered chart employing RSS, MRSS, and ERSS are 36.50, 31.73, and 41.91. Similarly, for the same shift δ = 0.20 and a different value of α, the ARL values are 50.89 for RSS, 43.63 for MRSS, and 52.45 for ERSS.
- Moreover, the ARL values utilizing LLF for suggested Bayesian chart under RSS designs are presented in Tables 5 and 6, the results designate that the ARL values for recommended Bayesian AEWMA under RSS at $ARL_{0} = 370$, δ = 0.20 and smoothing constant $\psi = 0.10$ is 7.42 and the ARL results at $\psi = 0.25$ is 12.42, in the same situation the ARL values under MRSS are 5.92 and 9.87. The ARL values using ERSS are 8.46 and 13.82. The results presented in Tables 3, 4, 5, and 6 demonstrate consistent efficiency for P and PP distributions under LLF.

**Table 1:** Run length results for the suggested chart applying SELF, for $\psi$ = 0.10, *n* = 5.

Shift	Bye-SRS EWMA		Bye-SRS AEWMA		Bye-RSS AEWMA		Bye-MRSS AEWMA		Bye-ERSS AEWMA	
	ARL	SDRL	ARL	SDRL	ARL	SDRL	ARL	SDRL	ARL	SDRL
	*L* = 2.7042		*h* = 0.0856		*h* = 0.00761		*h* = 0.00541		*h* = 0.00910	
0.00	371.82	367.80	372.86	537.77	370.25	423.48	371.55	424.82	370.13	457.80
0.20	125.58	114.98	70.61	91.12	38.40	35.95	32.19	29.64	41.80	39.45
0.30	66.576	57.92	35.40	44.53	11.56	10.83	15.58	14.94	21.96	21.07
0.40	41.68	32.78	21.15	26.36	19.19	18.46	9.13	8.81	12.87	12.40
0.50	28.35	20.12	13.55	16.69	7.41	6.90	5.94	5.47	8.30	7.93
0.60	20.98	13.49	9.46	11.23	5.26	4.83	4.20	3.60	5.94	5.52
0.70	16.26	9.59	7.08	7.70	3.93	3.43	3.14	2.58	4.43	3.92
0.75	14.72	8.30	6.15	6.43	3.51	2.93	2.87	2.29	3.89	3.36
0.80	13.41	7.14	5.62	5.82	3.10	2.54	2.57	1.99	3.47	2.93
0.90	11.42	5.69	4.51	4.18	2.55	1.92	2.08	1.47	2.86	2.27
1.00	9.78	4.50	3.85	3.20	2.19	1.56	1.80	1.16	2.39	1.80
1.50	5.79	2.03	2.25	1.29	1.28	0.58	1.16	0.42	1.37	0.67
2.00	4.16	1.20	1.66	0.78	1.06	0.25	1.02	0.15	1.09	0.30
2.50	3.31	0.84	1.36	0.56	1.00	0.08	1	0	1.01	0.12
3.00	2.76	0.66	1.17	0.39	1	0	1	0	1	0
4.00	2.12	0.38	1.02	0.14	1	0	1	0	1	0

**Table 2:** ARL outcomes for the offered chart using Bayesian approach given SELF, for $\psi$ = 0.10, *n* = 5.

Shift	Bye-SRS EWMA		Bye-SRS AEWMA		Bye-RSS AEWMA		Bye-MRSS AEWMA		Bye-ERSS AEWMA	
	ARL	SDRL	ARL	SDRL	ARL	ARL	SDRL	ARL	SDRL	ARL
	*L* = 2.8987		*h* = *h* = 0.242		*h* = 0.0184		*h* = 0.0128		*h* = 0.0158	
0.00	369.49	364.82	369.00	367.39	369.23	360.00	370.03	364.48	370.32	317.65
0.20	178.20	175.14	97.04	80.91	49.08	32.79	44.00	28.28	54.21	35.50
0.30	104.70	100.95	55.71	42.80	27.63	18.49	15.65	11.14	31.22	20.64
0.40	63.11	58.20	36.15	25.09	17.42	11.91	14.99	10.30	20.09	13.68
0.50	41.21	36.61	25.95	17.04	11.95	8.36	9.77	6.89	13.73	9.66
0.60	28.45	24.57	19.80	12.20	8.51	6.03	7.01	4.93	9.78	6.83
0.70	20.61	16.37	15.41	9.09	6.26	4.45	5.21	3.68	7.41	5.28
0.75	17.97	13.87	14.11	8.17	5.50	3.92	4.54	3.15	6.51	4.58
0.80	15.71	11.75	12.87	7.26	4.88	3.41	4.05	2.80	5.72	4.03
0.90	12.51	8.86	10.76	5.97	3.92	2.71	3.22	2.15	4.61	3.15
1.00	10.22	6.77	9.17	4.96	3.22	2.17	2.66	1.72	3.73	2.47
1.50	5.15	2.51	4.90	2.77	1.66	0.89	1.43	0.69	1.85	1.04
2.00	3.46	1.33	2.98	1.83	1.18	0.42	1.08	0.28	1.28	0.54
2.50	2.66	0.86	1.98	1.15	1.03	0.19	1	0	1.07	0.27
3.00	2.19	0.61	1.48	0.72	1	0	1	0	1	0
4.00	1.66	0.50	1	0	1	0	1	0	1	0

**Table 3:** ARL and SDRL values of the chart utilizing LLF, for $\psi$ = 0.10, *n* = 5.

Shift	Bye-SRS EWMA		Bye-SRS AEWMA		Bye-RSS AEWMA		Bye-MRSS AEWMA		Bye-ERSS AEWMA	
	ARL	SDRL	ARL	SDRL	ARL	ARL	SDRL	ARL	SDRL	ARL
	*L* = 2.7047		*h* = 0.086		*h* = 0.00772		*h* = 0.00539		*h* = 0.00924	
0.00	370.63	368.13	370.98	539.06	369.29	391.15	369.68	381.47	370.05	422.60
0.20	123.94	115.00	71.98	92.48	36.50	35.37	31.73	30.32	41.91	40.02
0.30	115.00	57.42	36.26	45.49	19.78	19.18	15.88	15.28	21.95	21.25
0.40	41.33	32.49	21.09	26.30	11.44	10.96	9.20	8.74	12.96	12.52
0.50	28.51	20.18	13.71	16.73	7.39	6.93	5.87	5.46	8.61	8.33
0.60	20.95	13.50	9.53	11.25	5.31	4.77	4.20	3.75	5.93	5.48
0.70	16.46	9.64	7.09	7.86	4.00	3.41	3.18	2.66	4.48	3.97
0.75	14.79	8.35	6.20	6.50	3.50	2.99	2.83	2.24	3.96	3.41
0.80	13.38	7.17	5.54	5.54	3.14	2.56	2.53	1.95	3.50	2.99
0.90	11.29	5.57	4.52	4.17	2.59	2.01	2.10	1.48	2.83	2.24
1.00	9.79	4.49	3.83	3.20	2.14	1.55	1.78	1.14	2.44	1.85
1.50	5.82	2.03	2.26	1.27	1.29	0.58	1.16	0.43	1.38	0.70
2.00	4.18	1.20	1.66	0.78	1.06	0.26	1.02	0.15	1.09	0.31
2.50	3.31	0.84	1.34	0.55	1.01	0.10	1	0	1.01	0.12
3.00	2.75	0.66	1.16	0.39	1	0	1	0	1	0
4.00	2.13	0.383	1.02	0.15	1	0	1	0	1	0

**Table 4:** Run length results using LLF for the proposed control chart with $\psi$ = 0.10, *n* = 5.

Shift	Bye-SRS EWMA		Bye-SRS AEWMA		Bye-RSS AEWMA		Bye-MRSS AEWMA		Bye-ERSS AEWMA	
	ARL	SDRL	ARL	SDRL	ARL	ARL	SDRL	ARL	SDRL	ARL
	*L* = 2.9050		*h* = 0.241		*h* = 0.0177		*h* = 0.0125		*h* = 0.0215	
0.00	371.05	368.88	370.14	434.88	370.92	332.68	369.44	344.38	371.12	316.71
0.20	179.81	175.30	86.77	83.25	50.89	32.73	43.63	28.65	52.45	35.89
0.30	105.54	101.21	55.44	42.26	29.15	19.09	23.39	15.70	31.99	20.90
0.40	64.00	59.50	36.76	25.98	18.11	12.29	14.72	10.26	20.49	13.77
0.50	41.56	37.30	25.86	16.88	12.37	8.59	9.81	6.98	13.79	9.59
0.60	28.54	24.33	19.65	12.16	8.64	6.10	6.91	4.87	10.00	6.95
0.70	20.96	16.82	15.62	9.17	6.56	4.59	5.13	3.66	7.52	5.28
0.75	18.11	14.02	14.23	8.29	5.71	4.02	4.52	3.16	6.60	4.57
0.80	15.89	11.94	12.83	7.30	5.10	3.58	4.03	2.77	5.74	4.04
0.90	12.61	8.89	10.79	5.90	4.01	2.72	3.23	2.17	4.62	3.19
1.00	10.27	6.77	9.25	5.00	3.36	2.20	2.65	1.74	3.81	2.56
1.50	5.18	2.50	4.95	2.80	1.69	0.91	1.42	0.67	1.88	1.06
2.00	3.46	1.33	2.97	1.81	1.19	0.43	1.09	0.30	1.28	0.53
2.50	2.64	0.85	1.97	1.13	1.04	0.20	1	0	1.07	0.27
3.00	2.19	0.62	1.48	0.73	1	0	1	0	1	0
4.00	1.66	0.50	1.09	0.30	1	0	1	0	1	0

**Table 5:** Using LLF, ARL and SRDL results of the Bayesian EWMA and AEWMA charts for PP distribution, for $\psi$ = 0.10, *n* = 5.

Shift	Bye-SRS EWMA		Bye-SRS AEWMA		Bye-RSS AEWMA		Bye-MRSS AEWMA		Bye-ERSS AEWMA	
	ARL	SDRL	ARL	SDRL	ARL	ARL	SDRL	ARL	SDRL	ARL
	*L* = 2.7018		*h* = 0.0856		*h* = 0.00763		*h* = 0.00533		*h* = 0.00919	
0.00	371.50	368.69	369.58	524.70	364.44	436.62	370.06	423.01	371.16	418.52
0.20	122.45	113.13	70.53	91.22	39.15	36.45	30.55	29.62	38.96	38.20
0.30	67.08	57.94	35.71	45.25	19.59	18.83	15.35	14.82	22.14	21.33
0.40	41.41	32.72	21.24	26.29	11.46	10.99	9.01	8.67	12.93	12.40
0.50	28.05	19.84	13.66	16.90	7.42	6.99	5.92	5.52	8.46	7.97
0.60	21.08	13.64	9.46	11.08	5.31	4.85	4.20	3.72	5.97	5.48
0.70	16.27	9.54	6.94	7.70	3.95	3.42	3.16	2.64	4.49	4.00
0.75	14.73	8.23	6.22	6.53	3.53	3.06	2.81	2.25	3.95	3.37
0.80	13.33	7.17	5.50	5.58	3.09	2.54	2.52	1.92	3.54	2.98
0.90	11.22	5.60	4.52	4.15	2.51	1.95	2.11	1.51	2.87	2.30
1.00	9.65	4.44	3.77	3.17	2.16	1.57	1.80	1.17	2.42	1.82
1.50	5.82	2.02	2.26	1.29	1.28	0.58	1.16	0.41	1.39	0.72
2.00	4.18	1.20	1.66	0.78	1.06	0.25	1.02	0.15	1.09	0.31
2.50	3.30	0.84	1.35	0.55	1	0.07	1	0	1.01	0.13
3.00	2.76	0.65	1.16	0.39	1	0	1	0	1	0
4.00	2.13	0.38	1.02	0.15	1	0	1	0	1	0

**Table 6:** Run length outcomes for the recommended chart with $\psi$ = 0.10, *n* = 5.

Shift	Bye-SRS EWMA		Bye-SRS AEWMA		Bye-RSS AEWMA		Bye-MRSS AEWMA		Bye-ERSS AEWMA	
	ARL	SDRL	ARL	SDRL	ARL	ARL	SDRL	ARL	SDRL	ARL
	*L* = 2.8986		*h* = 0.2414		*h* = 0.0179		*h* = 0.0127		*h* = 0.0212	
0.00	370.23	368.87	368.67	359.45	371.18	351.20	370.56	348.97	369.23	351.25
0.20	177.79	174.80	98.16	83.24	51.42	33.50	36.48	26.34	54.59	35.50
0.30	103.68	99.36	54.92	41.45	29.24	19.01	23.96	16.06	31.48	20.70
0.40	63.21	58.43	36.19	25.48	18.34	12.30	14.77	10.17	20.16	13.69
0.50	41.26	37.00	25.97	17.13	12.42	8.66	9.87	7.01	13.82	9.46
0.60	28.35	24.16	19.68	12.21	8.87	6.27	7.00	4.93	9.83	6.88
0.70	20.68	16.45	15.56	9.19	6.56	4.63	5.20	3.64	7.40	5.21
0.75	18.04	14.01	14.18	8.26	5.65	3.98	4.55	3.18	6.49	4.60
0.80	15.79	11.87	12.79	7.24	5.13	3.55	3.99	2.74	5.80	4.08
0.90	12.52	8.81	10.74	5.93	4.10	2.82	3.22	2.16	4.65	3.22
1.00	10.23	6.71	9.20	4.98	3.34	2.24	2.69	1.73	3.77	2.54
1.50	5.18	2.51	4.94	2.79	1.71	0.91	1.43	0.69	1.88	1.05
2.00	3.46	1.33	2.95	1.81	1.20	0.44	1.09	0.29	1.29	0.54
2.50	2.65	0.86	1.98	1.14	1.04	0.20	1	0	1.07	0.27
3.00	2.19	0.62	1.48	0.72	1	0	1	0	1	0
4.00	1.66	0.50	1.09	0.30	1	0	1	0	1	0

The suggested chart with Bayesian analysis under RSS designs for P and PP distributions, applying an informative prior and taking into account both LFs, namely SELF and LLF, are presented in Tables 1, 2, 3, 4, 5, and 6. The results show that, in comparison to existing RSS designs, the suggested chart applying MRSS has effective out-of-control signal identification.

## Real data applications

The practical demonstration of the suggested Bayesian method utilizing RSS designs utilizing various LFs under an informative prior is exemplified through an analysis of data from Montgomery^32^, focusing on the hard-bake process in semiconductor manufacturing. This particular process involves the application of heat to the photoresist material during the photolithography procedure to remove any residual solvents and ensure a uniform and stable surface for light exposure. Data was gathered from a semiconductor production facility, consisting of critical dimension measurements for wafers that underwent diverse hard-bake processes. Each set of data, totaling 45 samples, included measurements in microns at one-hour intervals for 5 wafers. Notably, the initial 30 samples were deemed to be under control, while the subsequent 15 samples were identified as out-of-control. We present a comprehensive description of the procedure for implementing the recommended AEWMA CC under RSS designs in the following steps.

i. First, classify your data into in-control and out-of-control observations. In your dataset, you have a total of 150 in-control observations and 75 out-of-control observations.
ii. For the in-control phase-I dataset (30 samples), perform RSS as follows:
  - From the in-control observations (150 in total), randomly select 25 observations. Divide the randomly selected 25 observations into 5 sets of 5 samples each, chosen randomly.
  - Within each set, arrange the 5 samples in ascending order, i.e., from the smallest to the largest
  - This step entails the selection of the initial value from the first set, the subsequent value from the second set, and so forth in a sequential manner.
  - This forms a ranked set sample of 5 values. d. Repeat steps (a-c) 30 times to create 30 in-control ranked set samples.
iii. Similar procedure is carried out for the out-of-control observations (75 in total) to choose the last 15 samples.
iv. Specify the informative prior for the Bayesian AEWMA control chart. In this example, the prior is taken as the standard normal distribution. Ensure that the properties of this prior distribution, such as mean and variance, are clearly defined.
v. Define the sampling distribution for the data. In our case, consider a normal distribution with specific parameters (mean and standard deviation). For instance, mean = 1.5043 and standard deviation = 0. 04,733,885. Compute the Bayes estimator $\hat{\theta }_{{\left( {LF} \right)RSS_{i} }}$.The Bayes estimator is standardized utilizing various LFs $y_{{\left( {LF} \right)}} = \frac{{\left( {\hat{\theta }_{{\left( {LF} \right)RSS_{i} }} - mean\left( {\left( {\hat{\theta }_{{\left( {LF} \right)RSS_{i} }} } \right)} \right)} \right)}}{{sd\left( {\left( {\hat{\theta }_{{\left( {LF} \right)RSS_{i} }} } \right)} \right)}}$, and computes the value of $h$ for fix $ARL_{0} = 370$.
vi. The subsequent 15 samples are designated as representing an out-of-control process, with the additional increment of 0.007 applied to each observation. These particular samples are then acknowledged as constituting a separate phase-II data set.
vii. Following both the methods and plot existing CC utilizing SRS and proposed CC.

**Figure 1:**
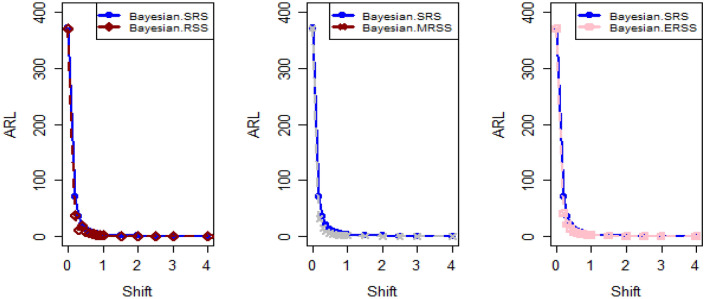
Applying SLEF, ARL plots of the suggested chart for P and PP distribution using various RSS strategies.

**Figure 2:**
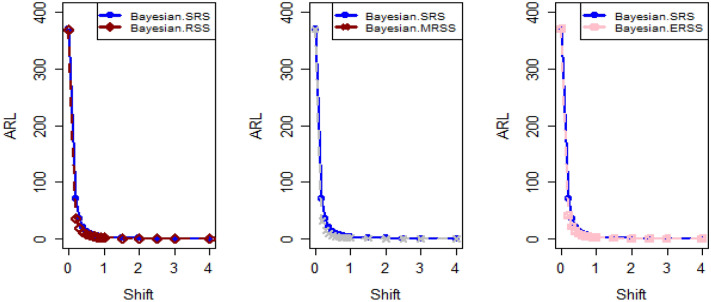
Based on LLF, ARL plots of the proposed chart for P distribution under different RSS designs.

**Figure 3:**
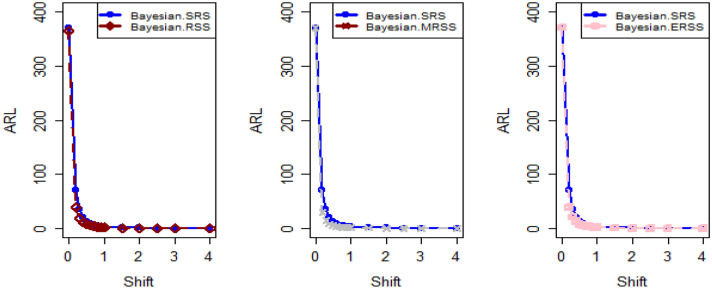
Using LLF, ARL plots of the proposed chart for PP distribution utilizing distinct RSS schemes.

**Figure 4:**
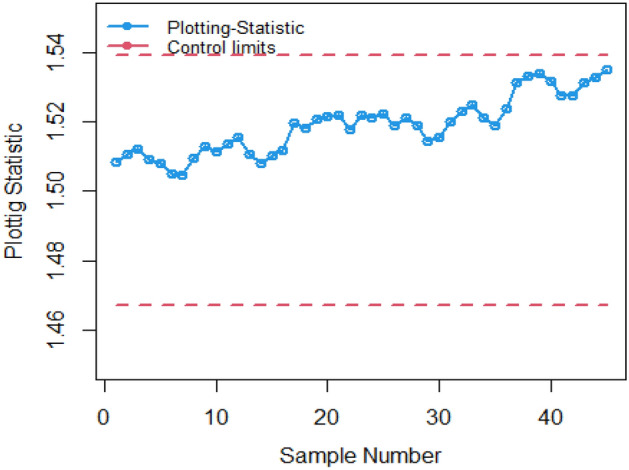
Applying SELF, Bayesian EWMA chart with SRS.

**Figure 5:**
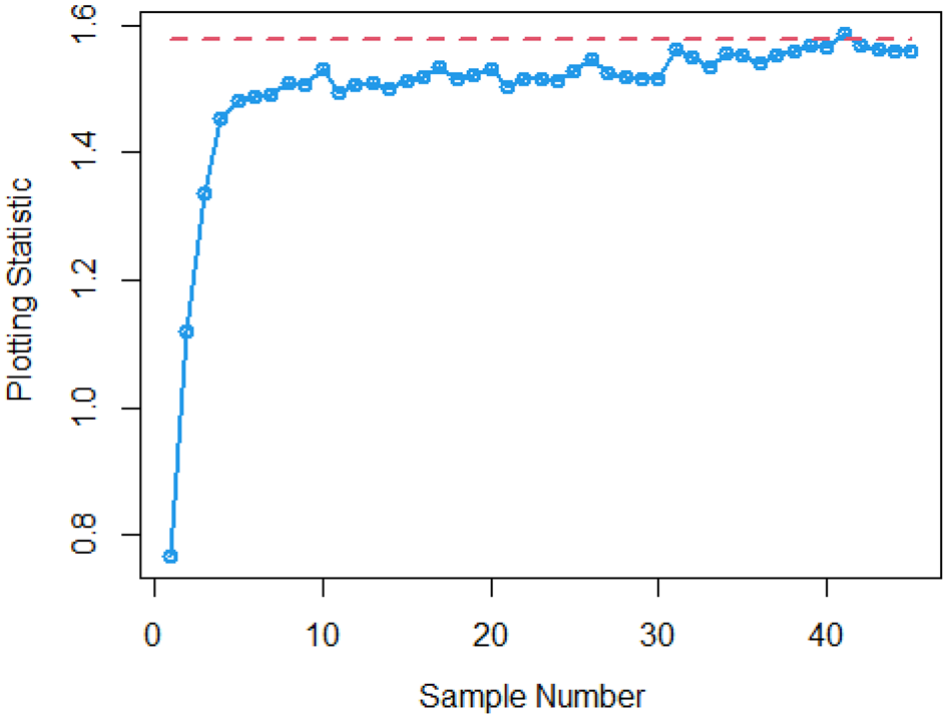
Based on SRS, plot for AEMWE chart utilizing SELF.

**Figure 6:**
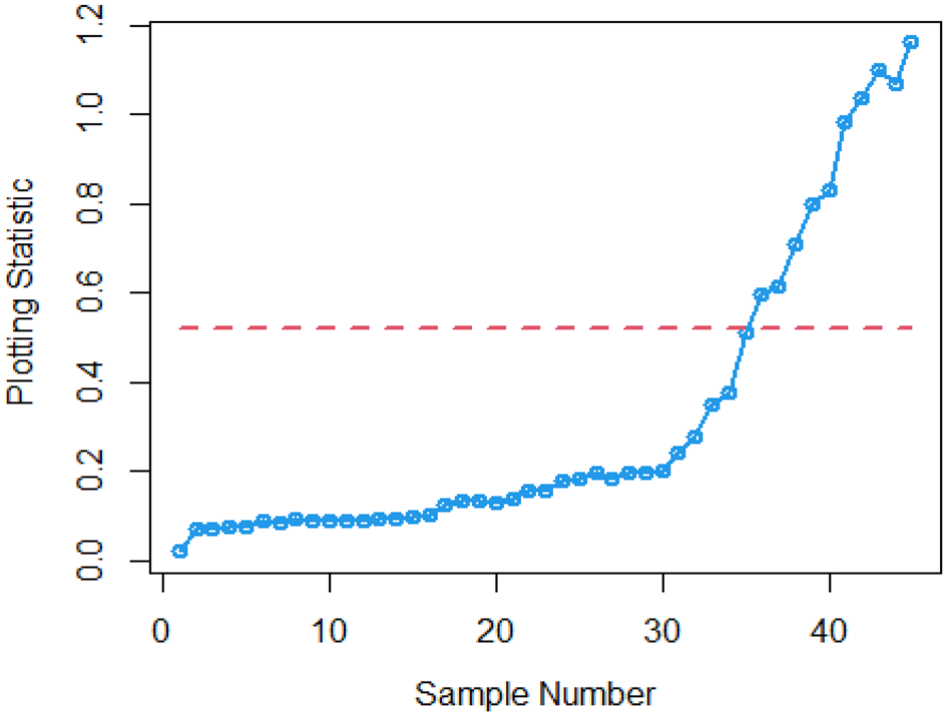
Applying RSS, plot of the suggested chart using P and PP distribution for SELF.

**Figure 7:**
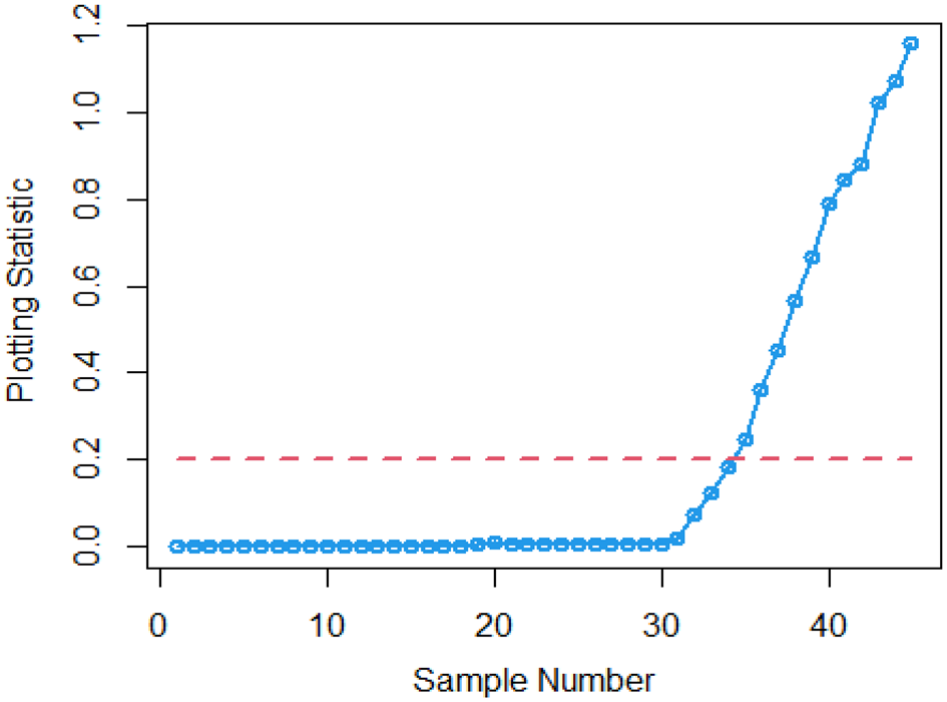
Based on MRSS, plot of proposed chart using for SELF.

**Figure 8:**
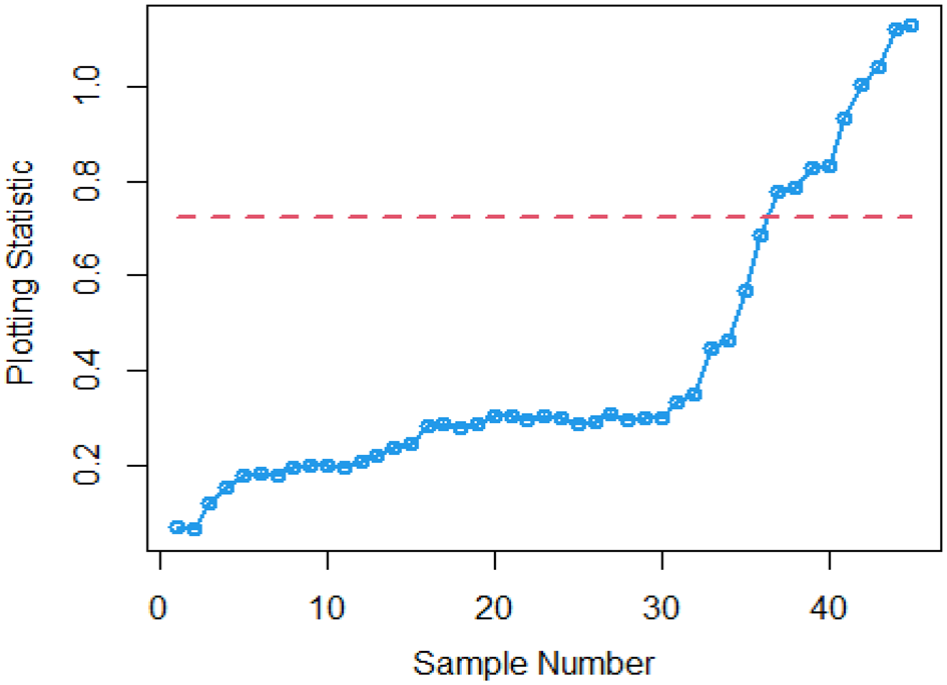
Under ERSS, plot of AEWMA chart using Bayesian approach based on SELF.

In Figs. 4 and 5, the existing Bayesian chart applying SRS under SELF are illustrated for P and PP distributions. The results show that all the points are in control for the EWMA chart, whereas for the AEWMA chart, out-of-control signals are detected starting from the 37th sample. Figures 6, 7, and 8 indicate the results of offered chart under SELF using RSS designs for P and PP distributions. The analysis of the figures indicates the detection of out of control signs on the 34th, 33nd, and 35th samples utilizing RSS, MRSS, and ERSS, respectively. Comparing Figs. 1, 2, 3, 4, 5, 6, 7, and 8, it becomes apparent that the suggested chart, implemented with RSS strategies, exhibits greater sensitivity in identifying out-of-control signs compared to the existing Bayesian charts implemented with SRS.

## Conclusion

This study presents a new Bayesian chart that utilizes various ranked set sampling strategies along with an informative prior. It incorporates two distinct LFs based on P and PP distributions for efficiently monitoring the process. The effectiveness of the suggested chart, implemented with RSS designs, is compared to an existing chart under Bayesian theory that uses SRS, as demonstrated in Tables 1, 2, 3, 4, 5, and 6. The ARL plots shown in Figs. 1, 2, and 3 further highlight the superior performance of the suggested chart. For evaluating its effectiveness across diverse RSS strategies, a numerical demonstration was conducted, employing data from the hard bake process in semiconductor production. The results indicated that the proposed chart exhibited improved efficiency in detecting out-of-control signals when compared to the EWMA and AEWMA charts implemented through the Bayesian theory with SRS.

## Limitations of the study

When dealing with large sample sizes, constructing the Bayesian AEWMA chart applying RSS designs can pose challenges. The process of Bayesian updating requires calculating the posterior distribution for both the process mean and variance at each sampled point, leading to significant time consumption and resource utilization. Moreover, establishing prior distributions for the process mean and variance within the Bayesian framework is a challenging task. Inaccurate or improperly chosen priors can substantially affect the control chart's performance. Furthermore, selecting suitable prior distributions often involves subjectivity, relying on expert knowledge, which could introduce bias into the analysis, further complicating the decision-making process.

## Future recommendation

The application of the recommended chart using RSS designs can be expanded to other charts that incorporate memory. Moreover, the suggested approach exhibits potential for accommodating distributions beyond the normal distribution. Certainly, the concept can be customized for various distributions like Binomial or Poisson distributions, requiring adjustments to the likelihood function for accurate estimations. Expanding the approach to cover diverse control charts and non-normal distributions can enhance quality control processes, benefiting sectors such as finance, healthcare, and production.

## Data Availability

This statement implies that the datasets utilized or examined during the ongoing research are accessible from the corresponding author upon a reasonable request. It underscores the author's willingness to provide access to the data for further examination or replication of the study's findings.
